# Trends in obesity prevalence and changes in adiposity across ethnic groups: findings from a population-based prospective cohort study in Amsterdam, the Netherlands (HELIUS study)

**DOI:** 10.1136/bmjph-2025-003837

**Published:** 2026-04-30

**Authors:** Yaw Kusi-Mensah, Esther M Vriend, Eric Moll van Charante, Henrike Galenkamp, Karien Stronks, Charles Hayfron-Benjamin, Sean Chetty, Didier Collard, Charles Agyemang, Bert-Jan H van den Born

**Affiliations:** 1 Amsterdam UMC, Amsterdam Public Health Research Institute, Department of Public & Occupational Health, University of Amsterdam, Amsterdam, The Netherlands; 2 Amsterdam UMC, Amsterdam Cardiovascular Sciences Institute, Department of Vascular Medicine, University of Amsterdam, Amsterdam, The Netherlands; 3 Department of Physiology, University of Ghana Medical School, Accra, Ghana; 4 Department of Anaesthesia and Critical Care, University of Ghana Medical School, Accra, Ghana; 5 Department of Anaesthesiology and Critical Care, Stellenbosch University Faculty of Medicine and Health Sciences, Cape Town, South Africa; 6 National Cardiothoracic Centre, Accra, Ghana; 7 Amsterdam UMC, Department of General Practice, University of Amsterdam, Amsterdam, The Netherlands; 8 Department of Medicine, Division of Endocrinology, Diabetes, and Metabolism, The Johns Hopkins University School of Medicine, Baltimore, Maryland, USA

**Keywords:** Public Health, Epidemiology, Body Mass Index, Cardiovascular Diseases, Obesity

## Abstract

**Background and aim:**

Obesity has reached epidemic proportions worldwide, with large differences across regions. European data on obesity trends among ethnic groups is scarce, particularly regarding the influence of age and socioeconomic status on these trends, which is crucial for guiding targeted interventions. In this study, we assessed the prevalence of obesity and changes in body mass index (BMI) and waist circumference (WC) over time and the effects of key sociodemographic factors on these changes using longitudinal data from a large multi-ethnic cohort.

**Methods:**

Using baseline and follow-up data from 10 484 participants in the HEalthy Life in an Urban Setting (HELIUS) study, representing Dutch, South-Asian Surinamese, African Surinamese, Ghanaian, Moroccan and Turkish populations; age-adjusted obesity prevalence; BMI and WC changes over an average follow-up of 6.3 (SD 1.2) years were calculated. Linear mixed models were used to assess changes in BMI and WC over time, adjusting for age, sex and socio-economic position. Key analyses were stratified by age (younger <50 years, older ≥50 years).

**Results:**

Median age of the baseline population was 48.0 years (IQR 38.0, 56.0), with 56.7% being women. Age-adjusted prevalence of obesity increased from 23.6% at baseline to 28.7% at follow-up, with steeper increases in ethnic minority groups compared with the Dutch origin group. The steepest increase in BMI and WC was observed in younger migrants, especially in those of Ghanaian and African-Surinamese descent. In older participants, only individuals of Ghanaian and Turkish descent showed a further increase in BMI over time.

**Conclusion:**

Obesity is increasingly prevalent in ethnic minority groups under 50 years, potentially raising obesity-related health risks and underscoring the need for targeted interventions to prevent further complications.

**WHAT IS ALREADY KNOWN ON THIS TOPIC**

- The increasing global burden of obesity and obesity-related diseases, coupled with its large regional and ethnic differences drives the various interventions implemented to mitigate the obesity epidemic. However, despite these efforts, obesity continues to increase in Europe and North America, where ethnic minority groups are largely impacted.

**WHAT THIS STUDY ADDS**

- This study highlights the increasing ethnic disparities in BMI, especially among young migrants, providing novel insights for improving interventions.

**HOW THIS STUDY MIGHT AFFECT RESEARCH, PRACTICE OR POLICY**

- This study necessitates further mechanistic research of obesity among migrants, the rethinking of previous intervention policies and the implementation of targeted obesity prevention strategies especially among young migrants.

## Introduction

The prevalence of obesity has increased significantly over the past few decades.^1^ In 2016, more than 1.9 billion adults were overweight, while over 650 million adults were described as obese^2^ according to the WHO. This trend is concerning because obesity is a major risk factor for non-communicable diseases (NCDs), including cardiometabolic disease (CMD), cancer and musculoskeletal disorders.^1 3^ Worldwide, there is considerable variation in obesity prevalence across geographical regions, and these geographical differences can be attributed to variations in ethnicity, migration patterns and socio-economic status,^4 5^ as well as their impact on lifestyle.^6–8^ Various interventions, including policy changes, community programmes and healthcare initiatives, have been implemented to mitigate the increasing obesity burden^9–11^; however, despite these efforts, obesity continues to increase.

Europe and North America are the two largest recipients of migrants from low-income countries,^12^ highlighting the need to assess ethnic differences in obesity prevalence over time in these regions, to guide the development of tailored interventions to mitigate the increasing obesity burden. At present, most data on obesity prevalence and incidence among various ethnic groups originate from the United States, while there is a paucity of data on the long-term prevalence of obesity among different ethnic groups in Europe. In addition, it remains unclear to what extent ethnic disparities in obesity extend to younger age groups. Therefore, we assessed changes in body mass index (BMI), waist circumference (WC), and prevalence of obesity over time among different ethnic groups, using longitudinal data from the HEalthy Life in an Urban Setting (HELIUS) study and explored the effects of age, sex and socioeconomic status on these changes.

## Materials and methods

### Study design, settings, study participants, sample size and ethical considerations

For this study, baseline and follow-up data of the HELIUS study were used. In brief, HELIUS is a large-scale, multi-ethnic population-based prospective cohort study carried out in Amsterdam, the Netherlands, which included 24 780 individuals between 2011 and 2015. At baseline, people aged 18–70 years were randomly sampled from the municipal registry with preferential sampling for the largest ethnic groups to allow for equal group sizes to facilitate comparisons between individuals of Dutch, Ghanaian, African and South-Asian Surinamese, Moroccan and Turkish descent. Participants received a written invitation and a response card. After a positive response, they received a confirmation of an appointment for a physical examination and a digital or paper version of the questionnaire. Non-Dutch ethnicity was based on the participant being born outside the Netherlands with at least one parent born in the same country (first generation) or being born in the Netherlands but with both parents born outside the Netherlands (migrants’ offspring, also called second generation). After data collection, the distinction between African Surinamese and South-Asian Surinamese participants was made based on self-report. For the Dutch sample, participants who were born in the Netherlands and whose parents were born in the Netherlands were invited. All persons who participated in the baseline examination and were still alive and living in the Netherlands received a written invitation for the follow-up examination (n=23 918). However, for the current analysis, participants with other/unknown ethnicity or missing BMI measurements at baseline were further excluded (n=2613), leaving 21 305 eligible participants. Follow-up data collection took place between 2019 and 2022. Data collection both at baseline and at follow-up consisted of a questionnaire/interview, a physical examination and the collection of biological samples. The HELIUS study was performed in compliance with the Declaration of Helsinki and has been approved by the Ethical Review Board of the Amsterdam UMC, location AMC, ethics number MREC 10/100# 17.10.1729. Written informed consent was provided by all participants. The HELIUS data is owned by the Amsterdam University Medical Centres, located in AMC, Amsterdam, The Netherlands. Any researcher can request the data by submitting a proposal to the HELIUS Executive Board as outlined at http://www.heliusstudy.nl/en/researchers/collaboration or by email: heliuscoordinator@amsterdamumc.nl. The HELIUS Executive Board will check proposals for compatibility with the general objectives, ethical approvals and informed consent forms of the HELIUS study. There are no other restrictions to obtaining the data and all data requests will be processed in the same manner. For further details, the rationale, conceptual framework, design and methods of the HELIUS study have been extensively described elsewhere.^13 14^

### Measurement of variables and definitions

Age and sex were obtained from the municipal registry, and other demographic information, medical history and lifestyle factors were obtained through a structured questionnaire using validated instruments. Smoking was defined based on self-report and was categorised as currently smoking or non-smoker. At both baseline and follow-up, weight in kilograms and height in metres were measured in light clothing without shoes using SECA 877 weighing scales and SECA 217 portable stadiometers, respectively (manufactured by SECA, Hamburg, Germany). BMI (kg/m^2^) was calculated by dividing the weight in kilograms by the squared height in metres. WC (cm) and hip circumference (cm) were measured using a standard measuring tape. For WC, the measurement was taken at the midpoint between the lower rib and the upper margin of the iliac crest, and hip circumference was measured around the major trochanter. Waist-to-hip ratio (WHR) was calculated by dividing WC by hip circumference. All anthropometric measurements were performed twice, and the average of the two measurements was used for the analysis. Office blood pressure (BP), defined as BP measured in a clinical setting by trained staff using standardised procedures, was taken twice on the left arm, at baseline and at the follow-up visit after at least 5 min rest while seated using the same validated semi-automatic oscillometric device (Microlife WatchBP Home; Microlife AG, Switzerland). The average of the two measurements at each visit was taken to denote systolic blood pressure (SBP) and diastolic blood pressure (DBP). Fasting blood samples were used to determine the concentration of glucose, total cholesterol and creatinine, while eGFR was calculated using the revised CKD-EPI formula 2021. Participants were asked to bring their prescribed medications, which were coded according to the Anatomical Therapeutic Chemical (ATC) classification.

Guided by the WHO classification, general obesity was defined as a BMI of 30 kg/m^2^ or more in Dutch, African Surinamese, Turkish, Ghanaian and Moroccan origin participants, and 27.5 kg/m^2^ or more in South-Asian Surinamese.^15^ Similarly, based on the WHO classification, central obesity was defined as WC ≥ 102 cm for South Asian-Surinamese men, ≥ 95cm for non South Asian-Surinamese men, and ≥ 88cm in women independent of ethnicity.^16^ ^17^ The different cut-offs for general obesity was used to ensure ethnic specificity as much as practical. Hypertension was defined as self-reported hypertension and/or the use of anti-hypertensive medication and/or increased blood pressure (BP) levels (SBP >140 mmHg and/or DBP >90 mmHg) according to current guidelines.^18^ Diabetes mellitus was defined as self-reported diabetes and/or use of a hypoglycaemic agent and/or fasting blood glucose >7 mmol/L and/or HBA1C >48 mmol/mol or >6.5%. Educational level, occupational status and occupational level were used as indicators for socioeconomic position (SEP). Highest educational level attained in The Netherlands or in the country of origin was measured at baseline and classified into four different categories: never been to school or elementary school, lower vocational schooling or lower secondary schooling, intermediate vocational schooling or intermediate or higher secondary schooling, and higher vocational schooling or university. Occupational status was measured at both baseline and follow-up classified into the following four categories: employed, not in working population (retirees, housemaker, students or schooling people), unemployed and unfit for work (incapacitated), whereas occupational level measured at baseline was classified per Dutch Standard Occupational Classification system for 2010 into the following categories: elementary occupations, lower occupations, middle or secondary occupations and higher occupations. The different indicators of SEP, although expected to demonstrate collinearity, did not affect the relationship between SEP and the change in BMI and WC within the different ethnic minority groups. This was evaluated using each factor separately and together in the model (data not shown).

### Data analysis

Data analysis was conducted using R version 4.2.1 (Vienna, Austria). Baseline characteristics were summarised as percentages, means (SD) or medians (IQRs) and compared using appropriate statistical tests such as ANOVA and Mann-Whitney U test, stratified by ethnicity. Age-adjusted prevalence rates of obesity (general and central) at baseline and follow-up were computed for complete cases and depicted using bar charts. Age-standardisation (using 5 year age intervals) was achieved using the baseline age distribution of each ethnic group, with Dutch origin participants serving as the reference. Changes in BMI over time were evaluated using linear mixed models, with a random intercept term for individual participants and fixed effects for visit, age (as determined at each visit), sex, and ethnicity. An interaction term between ethnicity and visit (0=baseline, 1=follow up) was added to examine ethnic disparities in BMI changes relative to Dutch participants. Age and sex were included in the model to account for potential confounding due to differences in their distribution across ethnic groups. The mixed effects model was chosen to account for missing follow-up data by including data of all participants at baseline. To assess the robustness of our findings, we additionally modelled ethnic disparities in BMI over time using linear regression models with change in BMI as the dependent variable. These results were largely consistent with those from the linear mixed models (data not shown). In an extended model, educational level, occupational status and occupational level at baseline were further included as indicators for socio-economic position. Multicollinearity was assessed for the extended models using variance inflation factors (VIF), with values >5 indicating potential multicollinearity. In addition, we calculated the percentage of attenuation between the two models ((βmodel 1 - βmodel 2)/ βmodel 1 expressed as a percentage) for all ethnic minority groups to clearly visualise the effect of SEP on the changes in BMI and WC. Separate analyses were conducted for younger (< 50 years) and older (≥ 50 years) subgroups based on the identified inflection point in the non-linear relationship between age and BMI (online supplemental figure 1). To assess whether changes in BMI over time differed between men and women, we also conducted separate analyses by sex as a subgroup analyses. Ethnic disparities in WC were assessed using similar modelling approaches. Sensitivity analyses compared results from complete cases analysis with those obtained through imputation. Imputation was performed for participants without follow-up data using multivariate imputation by chained equations (MICE), with the following variables: age, sex, blood pressure levels, baseline BMI, estimated glomerular filtration rate (eGFR), smoking status, diabetes mellitus and follow-up time. All statistical tests were two-sided with a significance level set at 0.05. Further information regarding the specific models can be found in the online supplemental material.

10.1136/bmjph-2025-003837.supp1 Supplementary data

### Patient and public involvement

None.

## Results

### Selection of participants

At baseline, 24 780 participants were included, and before the follow-up data collection, 313 participants were deceased and 549 had moved abroad and thus were not eligible. Of the 23 918 eligible follow-up participants, 6501 declined participation and 4315 were not reachable. Participants with other/unknown ethnicity or missing BMI at baseline (n=2613) and missing BMI at follow-up (n=5) were further excluded. In total, 10 489 participants had complete data regarding BMI and waist and hip circumference at baseline and follow-up (online supplemental figure 2). The mean follow-up time was 6.3 years (SD 1.2).

### Baseline characteristics of the included population

Details of all baseline characteristics stratified by ethnicity are shown in table 1, while baseline characteristics of the included participants, stratified by the availability of follow-up data and ethnicity, can be found in online supplemental table 1. At baseline, the median age was 48 years (IQR 38.0–56.0) and 56.6% of the participants were women. In total, 23.7% of participants had general obesity, whereas 64.5% had central obesity. The average BMI and WC of the study participants were 26.7 kg/m^2^ (SD 4.8) and 91.8 cm (SD 12.7), respectively. Compared with those without follow-up data, participants with follow-up were slightly older at baseline and generally healthier, a pattern that was broadly similar across ethnic groups.

**Table 1:** Baseline characteristics of the included population, stratified by ethnicity

	Dutch n=2992	South-Asian Surinamese n=1730	African Surinamese n=2130	Ghanaian n=901	Turkish n=1099	Moroccan n=1632
Demographics						
Age (years), median (IQR)	49.0 (38.0, 58.0)	48.0 (39.0, 56.0)	52.0 (43.0, 58.0)	47.0 (40.0, 53.0)	43.0 (33.0, 50.0)	43.0 (34.0, 51.0)
Sex (women), n (%)	1568 (52.4)	982 (56.8)	1354 (63.6)	544 (60.4)	574 (52.2)	914 (56.0)
Hypertension, n (%)	741 (24.8)	626 (36.2)	983 (46.2)	457 (50.9)	254 (23.1)	329 (20.2)
Current smoker, n (%)	654 (21.9)	406 (23.6)	587 (27.7)	42 (5.0)	313 (29.4)	161 (10.2)
Diabetes mellitus, n (%)	87 (2.9)	264 (15.3)	191 (9.0)	65 (7.2)	70 (6.4)	148 (9.1)
Lipid profile						
Total cholesterol level (mmol/l), mean (SD)*	5.2 (1.1)	5.0 (1.0)	4.9 (1.0)	4.9 (1.0)	4.9 (1.0)	4.7 (0.9)
Lipid-lowering medication, n (%)	205 (6.9)	339 (19.6)	199 (9.3)	50 (5.5)	81 (7.4)	107 (6.6)
eGFR (ml/min/1.73 m^2^), mean (SD)	93.6 (14.9)	96.2 (15.8)	101.2 (18.1)	102.1 (18.8)	106.1 (13.6)	107.5 (14.3)
History of CVD, n (%)	36 (1.2)	36 (2.1)	44 (2.1)	8 (1.0)	13 (1.2)	12 (0.8)
Educational level, n (%)						
Never	75 (2.5)	222 (12.9)	91 (4.3)	237 (28.6)	274 (25.8)	469 (29.8)
Lower	368 (12.4)	565 (32.8)	705 (33.4)	330 (39.9)	250 (23.6)	301 (19.1)
Intermediate	641 (21.6)	505 (29.3)	753 (35.6)	203 (24.5)	314 (29.6)	497 (31.6)
Higher	1886 (63.5)	430 (25.0)	564 (26.7)	58 (7.0)	222 (20.9)	308 (19.6)
Occupational status, n (%)						
Employed	2291 (76.9)	1134 (66.2)	1471 (69.5)	515 (62.5)	628 (59.5)	861 (54.8)
Not in working population	470 (15.8)	209 (12.2)	224 (10.6)	40 (4.9)	192 (18.2)	362 (23.0)
Unemployed	140 (4.7)	218 (12.7)	274 (12.9)	203 (24.6)	156 (14.8)	226 (14.4)
Incapacitated	78 (2.6)	152 (8.9)	147 (6.9)	66 (8.0)	80 (7.6)	123 (7.8)
Occupational level, n (%)						
Elementary	43 (1.5)	149 (9.7)	110 (5.6)	447 (63.6)	147 (17.0)	203 (17.1)
Lower	378 (13.3)	488 (31.8)	635 (32.3)	164 (23.3)	316 (36.5)	393 (33.1)
Medium	653 (23.0)	499 (32.5)	703 (35.8)	62 (8.8)	220 (25.4)	350 (29.5)
Higher	1768 (62.2)	398 (25.9)	516 (26.3)	30 (4.3)	183 (21.1)	242 (20.4)
Body composition						
BMI (kg/m^2^), mean (SD)	24.7 (4.0)	26.2 (4.6)	27.7 (5.2)	28.3 (4.4)	28.0 (5.2)	27.6 (4.8)
Waist-to-hip ratio, mean (SD)	0.9 (0.1)	0.9 (0.1)	0.9 (0.1)	0.9 (0.1)	0.9 (0.1)	0.9 (0.1)
Waist circumference (cm), mean (SD)	89.4 (12.3)	91.5 (12.4)	92.8 (13.1)	92.8 (11.4)	93.4 (13.1)	93.9 (12.6)
Obesity, n (%)	265 (8.9)	572 (33.1)	595 (27.9)	291 (32.3)	323 (29.4)	440 (27.0)
Central obesity, n (%)	1610 (53.8)	1237 (71.6)	1415 (66.5)	625 (69.4)	741 (67.4)	1127 (69.1)

Obesity was defined as a BMI of ≥27.5 kg/m2 for the South-Asian Surinamese and ≥30 kg/m2 for the other ethnic groups. Central obesity was defined as a waist circumference of ≥88 cm for women independent of ethnicity, ≥102 cm for South-Asian Surinamese men and ≥95 cm for non-South-Asian Surinamese men. Hypertension was defined as RR ≥140/90 mmHg or use of antihypertensive medication. Diabetes mellitus was defined as glucose ≥7 mmol/l or use of glucose-lowering medication. History of CVD was defined as self-reported stroke, myocardial infarction, and coronary or peripheral revascularization.

BMI, body mass index; CVD, cardiovascular diseases; eGFR, estimated glomerular filtration rate.

At follow-up, the percentage of participants with general obesity increased to 28.7%, while 66.8% of all participants had central obesity. Average BMI and WC levels increased to 27.3 kg/m2 (SD 5.0) and 92.6 cm (SD 12.9), respectively.

### Change in prevalence of central and general obesity over time

**Figure 1:**
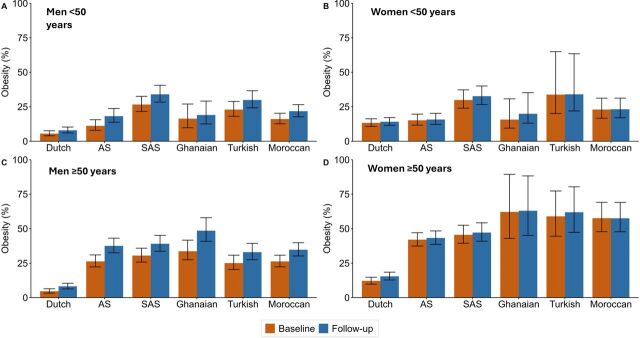
Age-adjusted prevalence of general obesity stratified by age group (<50 (A & B) vs ≥50 (C & D) years), sex and ethnicity. Age-standardisation (using 5 year age intervals) was achieved using the baseline age distribution of each ethnic group, with Dutch origin participants serving as the reference. Obesity was defined as a BMI of ≥27.5 kg/m^2^ for the South-Asian Surinamese and ≥30 kg/m^2^ for the other ethnic groups. AS, African Surinamese; BMI, body mass index; SAS, South-Asian Surinamese.

**Figure 2:**
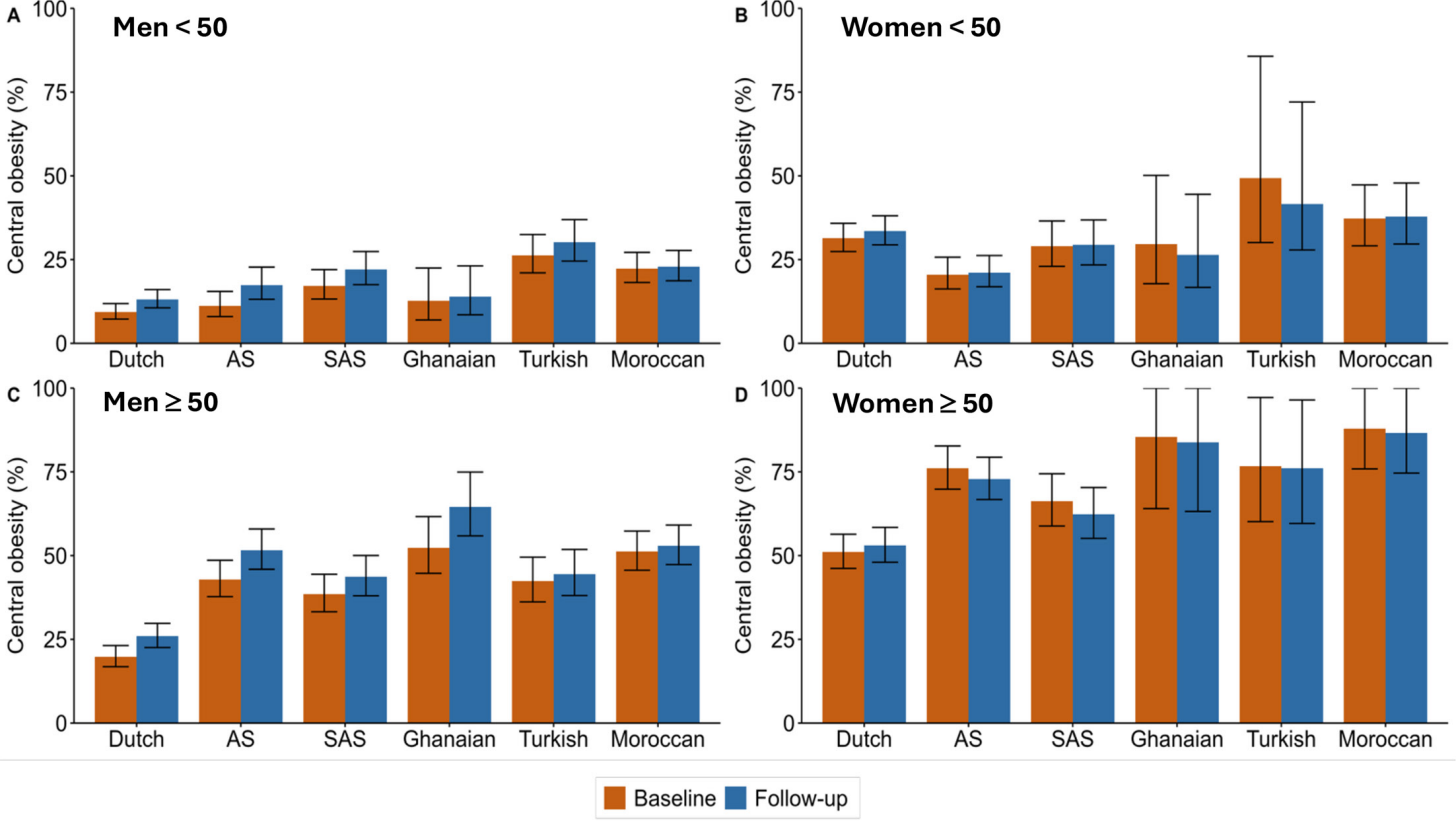
Age-adjusted prevalence of central obesity stratified by age group (<50 (A & B) vs ≥50 (C & D) years), sex and ethnicity. Age-standardisation (using 5 year age intervals) was achieved using the baseline age distribution of each ethnic group, with Dutch origin participants serving as the reference. Central obesity was defined as a waist circumference of ≥88 cm for women, ≥102 cm for South-Asian Surinamese men and ≥95 cm for non-South-Asian Surinamese men. AS, African Surinamese; SAS, South-Asian Surinamese.

Figures 1 and 2 show the age-adjusted prevalence rates of general obesity and central obesity for younger (<50 years) and older (≥50 years) participants stratified by sex. Although CIs were relatively wide due to age-adjustment and largely overlapped, we observed a trend towards increased prevalence of both general and central obesity over time among younger individuals from all ethnic minority groups, with the most pronounced increase in general obesity among Ghanaian participants, followed by the African-Surinamese and South-Asian Surinamese. In the older age group, the prevalence of general obesity remained relatively stable across ethnic groups, while central obesity appeared to decrease over time.

### Change in BMI and WC over time

Results from the linear mixed models are depicted in table 2, showing the beta for change in BMI over time for the different ethnic minority groups adjusted for age and sex (model 1) and for age, sex, educational level, occupational status and occupational level (model 2). In model 1, all ethnic minority groups had an additional increase in their BMI over time compared with the Dutch in the younger participants, with Ghanaians recording the highest additional increase in BMI (0.81 kg/m^2^, 95% CI 0.60 to 1.03, p<0.001). In the older participants, a statistically significant additional increase in BMI compared with the Dutch participants was only present in the Ghanaian (0.45 kg/m2, 95% CI 0.23 to 0.67, p<0.001) and the Turkish participants (0.46 kg/m^2^, 95% CI 0.23 to 0.69, p<0.001). In both the younger and older participants, further adjustment of educational level, occupational status and occupational level yielded comparable results. Full details of the linear mixed models with estimates for age and sex can be found in online supplemental table 2. Percentages of attenuation for ethnic differences in BMI when adjusting for SEP for ethnic minority groups are shown in online supplemental table 3. In the younger participants, SEP explained a small portion of the steeper additional change in BMI, particularly for Ghanaians (1.2%) and African-Surinamese (4.1%), while the effect of SEP on the change in BMI over time was more pronounced among the Moroccan (17.6%), Turkish (10.3%) and South-Asian Surinamese (7.9%) population. In the older participants however, the attenuation percentages were generally higher.

**Table 2:** Ethnic differences in change in BMI over time compared with the Dutch reference population as derived from linear mixed models

	Younger (< 50 years)								Older (≥ 50 years)							
	Model 1				Model 2				Model 1				Model 2			
	β	95% CI		P-value	β	95% CI		P-value	β	95% CI		P-value	β	95% CI		P-value
Difference in BMI between baseline and follow-up in Dutch reference population	0.61	0.60	0.62	<0.001	0.62	0.61	0.63	<0.001	0.14	0.13	0.14	<0.001	0.14	0.13	0.14	<0.001
Additional increase in BMI at follow-up compared with Dutch reference population																
Dutch	Ref	Ref	Ref	Ref	Ref	Ref	Ref	Ref	Ref	Ref	Ref	Ref	Ref	Ref	Ref	Ref
SA Surinamese	0.44	0.26	0.62	<0.001	0.45	0.26	0.63	<0.001	0.02	−0.14	0.19	0.773	0.08	−0.09	0.26	0.339
A Surinamese	0.54	0.36	0.73	<0.001	0.50	0.31	0.69	<0.001	0.08	−0.07	0.22	0.281	0.08	−0.07	0.23	0.292
Ghanaian	0.81	0.60	1.03	<0.001	0.78	0.54	1.01	<0.001	0.45	0.23	0.67	<0.001	0.44	0.20	0.69	<0.001
Turkish	0.34	0.15	0.53	<0.001	0.36	0.16	0.56	0.001	0.46	0.23	0.69	<0.001	0.51	0.25	0.78	<0.001
Moroccan	0.44	0.27	0.61	<0.001	0.38	0.20	0.56	<0.001	0.03	−0.16	0.22	0.760	0.14	−0.09	0.37	0.244

Model 1 was adjusted for age and sex. Model 2 was additionally adjusted for educational level, occupational status and occupational level.

A, African; BMI, body mass index; SA, South-Asian.

Table 3 shows the beta for change in WC over time for the different ethnic minority groups, adjusted for age and sex (model 1) and additionally adjusted for educational level, occupational status and occupational level (model 2). In the Dutch participants, the mean WC increased by 0.97 cm (95% CI 0.90 to 1.04, p<0.001) over time in participants under the age of 50 years, while it showed no significant change from baseline to follow-up in participants 50 years and above. In participants under 50 years, the South-Asian Surinamese, African-Surinamese and Ghanaians showed a significant steeper increase in WC over time compared with the Dutch reference group, with the highest additional increase in mean WC in the Ghanaian participants (1.47 cm, 95% CI 0.75 to 2.18, p<0.001). Additional adjustment for educational level, occupational status and occupational level did not materially change the results. Percentages of attenuation for ethnic differences in WC when adjusting for SEP for ethnic minority groups are shown in online supplemental table 4. In the younger participants, SEP explained a small portion of the additional change in WC, particularly for Ghanaians (4.8%) and African-Surinamese (4.0%), while the effect of SEP on the change in WC over time was more pronounced among the Moroccan (33.3%), Turkish (20.0%) and South-Asian Surinamese (7.2%) population. In the older participants however, the attenuation percentages were generally higher.

**Table 3:** Ethnic differences in change in waist circumference over time compared with the Dutch reference population as derived from linear mixed models

	Younger (< 50 years)								Older (≥ 50 years)							
	Model 1				Model 2				Model 1				Model 2			
	β	95% CI		P-value	β	95% CI		P-value	β	95% CI		P-value	β	95% CI		P-value
Difference in WC between baseline and follow-up in Dutch reference population	0.97	0.90	1.04	<0.001	0.98	0.90	1.05	<0.001	0.01	−0.05	0.07	0.743	0.05	−0.01	0.11	0.103
Additional increase in WC at follow-up compared with Dutch reference population																
Dutch	Ref	Ref	Ref	Ref	Ref	Ref	Ref	Ref	Ref	Ref	Ref	Ref	Ref	Ref	Ref	Ref
SA Surinamese	1.17	0.57	1.76	<0.001	0.77	0.16	1.38	0.014	−0.49	−1.06	0.08	0.089	−0.35	−0.94	0.24	0.248
A Surinamese	1.47	0.76	2.18	<0.001	1.20	0.59	1.81	<0.001	−0.23	−0.72	0.27	0.376	−0.28	−0.79	0.23	0.283
Ghanaian	−0.49	−1.11	0.13	0.123	1.30	0.52	2.07	0.001	0.49	−0.26	1.25	0.199	0.51	−0.31	1.34	0.223
Turkish	−0.09	−0.65	0.47	0.757	−0.20	−0.87	0.46	0.552	−0.73	−1.52	0.07	0.072	−0.57	−1.47	0.33	0.216
Moroccan	0.78	0.19	1.37	0.010	−0.01	−0.61	0.59	0.970	−0.58	−1.24	0.07	0.080	−0.21	−1.00	0.58	0.600

Model 1 was adjusted for age and sex. Model 2 was adjusted for age, sex, educational level, occupational status and occupational level.

A, African; SA, South-Asian; WC, waist circumference.

### Sex differences in the change in BMI and WC over time

There were significant interactions in the association of ethnicity with BMI and WC over time according to sex. Adjusted for age and socio-economic position, Dutch women aged <50 years showed a greater increase in BMI over time (0.75 kg/m², 95% CI 0.74 to 0.77, p<0.001) compared with Dutch men (0.46 kg/m², 95% CI 0.45 to 0.47, p<0.001), a pattern similarly observed in participants aged ≥50 years. In those aged <50 years, women of African-Surinamese descent had the highest additional increase in BMI (0.96 kg/m², 95% CI 0.63 to 1.29, p<0.001) over time compared with Dutch women (model 2), while Turkish men showed the highest BMI increase compared with Dutch men (0.51 kg/m², 95% CI 0.27 to 0.76, p<0.001). Among Dutch participants aged >50 years, men showed a greater increase in WC over time (0.99 cm, 95% CI 0.88 to 1.10, p<0.001) compared with Dutch women (0.93 cm, 95% CI 0.77 to 1.09, p<0.001) in models adjusted for age and socio-economic position. There were no significant additional changes in WC in the ethnic minority groups compared with the Dutch reference group, except in African-Surinamese (1.07 cm, 95% CI 0.20 to 1.94, p=0.015) and Ghanaian women (1.67 cm, 95% CI 0.59 to 2.74, p=0.002) and African-Surinamese men aged <50 years (1.44 cm, 95% CI 0.59 to 2.28, p=0.001). The sex-stratified analysis in BMI and WC of the different ethnic groups is shown in online supplemental figures 3 and 4. Results from the linear mixed models for changes in BMI and WC, stratified by sex and age, are also presented in online supplemental tables 5 and 6.

### Sensitivity analysis following imputation

Results from the sensitivity analysis following imputation for changes in BMI and WC over time are shown in online supplemental tables 7 and 8. In the participants under 50 years, the analysis post-imputation yielded results largely consistent with those obtained without imputation, although with a reduction in estimates. For the Turkish participants, the difference in change in BMI compared with the Dutch became non-significant (0.29 kg/m², 95% CI −0.07 to 0.64, p=0.109) after imputation. In the participants 50 years and above, ethnic disparities in BMI level changes compared with the Dutch disappeared after imputation.

## Discussion

In this multi-ethnic population study, we show that both general and central obesity increased over time, especially among ethnic minority groups under the age of 50. Younger migrants, especially those of Ghanaian and African-Surinamese descent, experienced the sharpest increases in BMI and WC, with socio-economic position not fully explaining this trend in ethnic minority groups. Stratification by sex revealed that Ghanaian women demonstrated the most notable increase in BMI compared with men, while there were no significant differences over time across other ethnic minority groups.

Globally, the prevalence of obesity has tripled between 1975 and 2016.^2^ In Europe, 52.7% of all adults aged 18 years and above are classified as overweight.^19^ Similarly, in the Netherlands, obesity prevalence has also tripled over the past 40 years, with around 16% of the population aged 20 years and above now considered to be living with obesity.^20 21^ The increasing prevalence of obesity is driven by nutritional factors and non-nutritional factors and persists despite policies and interventions aimed at mitigating obesity.^2^ In addition, the steeper increase over time in the age-adjusted prevalence of general obesity and, to a lesser extent, central obesity among different ethnic minority groups is comparable to prior cross-sectional studies in the US, UK, and Australasia, where different ethnic minority groups have a higher prevalence of individuals living with obesity compared with the host population.^22–24^ In North America, for example, Hispanic and African American individuals have higher rates of overweight and obesity compared with non-Hispanic Whites,^24^ while in the UK, higher rates of obesity are observed in South-Asian, African, and Caribbean populations compared with the general populations.^25^

The age-related differences in obesity prevalence and the changes in BMI and WC over time are in accordance with the findings of a recent pooled analysis of global trends in underweight and obesity from 1990 to 2022, which revealed a shift in the onset of obesity to younger age categories.^26^ This shift explains the steeper increase in obesity prevalence in younger populations. Further, this shift to younger populations is also supported by the higher prevalence of childhood and adolescent obesity. Globally, the prevalence of overweight (including obesity) in children between the ages of 5 and 19 has increased from 8% in 1990 to 20% in 2022, with a quadrupling of the obesity prevalence in adolescents alone.^27^ Another possible explanation for the observed age-related differences in BMI changes over time could be a shift towards a more sedentary lifestyle among younger populations, potentially exacerbated by the COVID-19 (SARS-CoV-2) pandemic, during which many people had to stay home and had limited social and work-related activities.^28^

Our findings show a steeper increase in obesity prevalence and BMI in the ethnic minority group under 50 years compared with the Dutch reference group. One possible explanation for the steeper increase in BMI among ethnic minority group under 50, compared with the Dutch reference population, is the socio-economic disparities across the different groups. However, after adjusting for educational level and occupational status at baseline, indirect measures of socio-economic position, the significant ethnic differences in change in BMI remained, suggesting the likelihood that socio-economic position does not fully explain these differences. A possible explanation for the observed differences is a shift towards Western-style diets that are high in calorie intake and saturated fats in migrants’ offspring rather than the more traditional and often healthier foods from the country of origin of their parents.^29 30^ This may also explain why older migrants might have no increase in central obesity over time, despite the slight increase in BMI among Turkish and Ghanaian descent populations.

We observed significant differences between men and women in changes in BMI and WC over time across all ethnic groups. Women had a higher increase in BMI, while men had a higher increase in WC. These findings are consistent with previous studies including Sanchez-Romero L. *et al*,^31^ and may be explained by well-documented differences in fat storage.^32–34^ In our study, Ghanaian women had the most notable increase in BMI. This may be attributable to differences in occupation activity and sociocultural perception of obesity, where weight gain is seen as a sign of prosperity and fertility. Additionally, dietary differences between sexes may play a role, with women consuming more sugar-laden foods and having a greater increase in triglycerides following refined sugar metabolism.^35^

Genetic background and gene-environment interactions, including epigenetic changes and gut microbiome variations, influence the propensity to gain weight and have been linked to the causal pathway for obesity, possibly explaining some of the differences between ethnic groups. Extensive research is ongoing regarding the role of the gut microbiome in obesity-related diseases to aid our understanding of ethnic differences in obesity-related disease burdens. Recently published HELIUS data has shown domination of Western-lifestyle associated bacterial clusters among Surinamese populations, while Moroccan and Turkish descent populations show a transition towards a low-diversity, less fermentatively capable gut microbiome.^30^ Notwithstanding the need for further evidence to establish causality, adaptations in the gut microbiome and its metabolites may further contribute to the observed differences in obesity prevalence and BMI changes.^36^ However, these mechanisms were not explored directly in this study, limiting the ability to establish a strong association between them and the trend in obesity indices observed. Further research is needed to study the changes in diets over time, the effects of age and gut microbiome on these changes, and the impact of dietary modifications on BMI and the obesity prevalence.

### Strengths and limitations

Strengths of the HELIUS study include the availability of substantial longitudinal data on the largest migrant groups in Europe from outside the European Union at the time of data collection, as well as a standardised approach to data collection among participants over time. A key limitation of this study was the loss to follow-up, resulting in an overall response rate of 46.4%, with major/large differences in response rates among the various ethnic groups. However, these differences did not lead to major discrepancies in representativeness.^14^ Previous analyses have shown that participants with healthier lifestyles at baseline are likely to participate in follow-up, creating a selection effect towards healthier study participants at follow-up. Given this selectivity, we would expect an underestimation of the BMI changes. Moreover, no selection effects based on ethnicity were observed, suggesting that the widening ethnic inequalities are less likely to be attributed to differential selection effects across ethnic groups. This assertion is somewhat supported by minimal changes in the effect estimates observed in most of the ethnic minority groups following imputation, although imputation has its limitations, especially in this setting where the type of missing data—whether MCAR or MAR or NMAR or SM—cannot be easily ascertained, and we assumed the MAR type. Another limitation of the HELIUS study is the fact that participant selection is ultimately based on one's willingness to participate in health research in general, suggesting that participants are more health conscious. However, we previously assessed differences between responders and non-responders at baseline and showed that responders were slightly older, more likely of Dutch and Surinamese descent, and had a higher socio-economic status.^37^ Given the association of these parameters with a higher prevalence of obesity among participants in HELIUS, this has likely resulted in an underestimation of the true prevalence of obesity at baseline in the population at large. Another limitation of the study is also the definition of socio-economic position and hence the conclusion in the regression model, where socio-economic position is adjusted for. Although educational level and occupational status indirectly measure one’s socio-economic position, a much more direct measure is income or wealth data, which was lacking in this study. Another limitation is that the follow-up period overlapped with the COVID-19 pandemic, which may have influenced participants’ willingness to participate in the study. However, we compared ethnic differences in BMI before and after the pandemic and found no significant differences.

## Conclusion

In this multi-ethnic cohort from Amsterdam, the prevalence of both general and central obesity, and obesity indices, such as BMI and WC, increased over time in all ethnic minority groups compared with the Dutch reference group, with this trend more pronounced in adults below 50 years of age. Crucially, the effect of SEP on this trend varied significantly among ethnic minority groups, underscoring the need for tailored preventive strategies, particularly for younger, second- (and probably also third-generation) migrants. The divergence in results for the Turkish and older cohorts in our sensitivity analysis suggests that these specific trends may be more sensitive to baseline health status or attrition. The present findings provide insights into the changes in the obesity epidemic in a multi-ethnic population and serve as a backdrop for further studies in evaluating the mechanisms driving obesity.

## Data Availability

Data are available upon reasonable request. The HELIUS data are owned by Amsterdam UMC, Amsterdam, The Netherlands. Any researcher can request the data by submitting a proposal to the HELIUS Executive Board as outlined at http://www.heliusstudy.nl/en/researchers/collaboration, by email: heliuscoordinator@amsterdamumc.nl. The HELIUS Executive Board will check proposals for compatibility with the general objectives, ethical approvals and informed consent forms of the HELIUS study. There are no other restrictions to obtaining the data and all data requests will be processed in the same manner.
