# Supplementary material for: Trends in obesity prevalence and changes in adiposity across ethnic groups: findings from a population-based prospective cohort study in Amsterdam, the Netherlands (HELIUS study)
Source: BMJ Public Health. 2026 Apr 30;4(2):e003837. doi: 10.1136/bmjph-2025-003837 (PMC13140977; doi:10.1136/bmjph-2025-003837)

Supplementary material

Statistical approach to assess change in BMI over time (replicated for WC)

The primary analysis of changes in BMI over time was performed using linear mixed-effects models.

The linear mixed model was specified as follows:

*lme(BMI ~ Ethnicity*Visit (0 = baseline, 1 = follow) + Sex + Age, random= ~1|Participant ID)*

Detailed explanation of the included variables:

- BMI is the BMI of the individuals at either the baseline or follow-up visit
- Ethnicity is a categorical variable indicating the ethnic group of participant
- Visit is a binary variable indicating the timepoint (0 = baseline, 1 = follow-up)
- Ethnicity*Visit is the interaction term testing whether changes in BMI over time differ between ethnic groups relative to the reference group (Dutch participants)
- Age and sex were included as fixed-effect covariates to control for potential confounding due to their varying distributions across ethnic groups.
- ~1|Participant ID is the random intercept term

Separate linear mixed models were run within predefined subgroups:

- Age subgroups:
  Participants were divided into two groups based on an identified inflection point in the nonlinear relationship between age and BMI:
  - Younger group: < 50 years
  - Older group: ≥ 50 years
- Sex subgroups:
  Separate analyses were conducted for men and women to assess whether the association between ethnicity and BMI change over time differed by sex.

We used similar models for change in waist circumference.

Supplementary Table 1: Baseline characteristics of the Dutch, South-Asian Surinamese, African Surinamese (A), and Ghanaian, Turkish, and Moroccan populations (B), stratified by the availability of follow-up data.

A		Dutch		South-Asian Surinamese		African Surinamese	
		Without follow-up	With follow-up	Without follow-up	With follow-up	Without follow-up	With follow-up
		n = 1598	*n = 2992*	*n = 1310*	*n = 1730*	*n = 2013*	*n = 2130*
Age (years), median [IQR]		41.0 [30.0, 57.0]	49.0 [38.0, 58.0]	45.0 [31.0, 55.8]	48.0 [39.0, 56.0]	49.0 [36.0, 56.0]	52.0 [43.0, 58.0]
Sex (women), n (%)		918 (57.4)	1568 (52.4)	688 (52.5)	982 (56.8)	1175 (58.4)	1354 (63.6)
Hypertension, n (%)		404 (25.4)	741 (24.8)	530 (40.6)	626 (36.2)	919 (45.8)	983 (46.2)
Current smoker, n (%)		475 (30.3)	654 (21.9)	453 (34.7)	406 (23.6)	719 (35.9)	587 (27.7)
Diabetes mellitus, n (%)		61 (3.9)	87 (2.9)	249 (19.1)	264 (15.3)	256 (12.9)	191 (9.0)
Lipid-lowering medication, n (%)		135 (8.4)	205 (6.9)	263 (20.1)	339 (19.6)	205 (10.2)	199 (9.3)
eGFR (ml/min/1.73 m^2), mean (SD)		97.2 (16.6)	93.6 (14.9)	98.0 (18.8)	96.2 (15.8)	104.3 (19.8)	101.2 (18.1)
History of CVD, n (%)		30 (1.9)	36 (1.2)	48 (3.7)	36 (2.1)	64 (3.2)	44 (2.1)
Educational level, n (%)	Never	75 (4.8)	75 (2.5)	214 (16.4)	222 (12.9)	139 (7.0)	91 (4.3)
	Lower	277 (17.7)	368 (12.4)	444 (34.1)	565 (32.8)	770 (38.6)	705 (33.4)
	Intermediate	353 (22.5)	641 (21.6)	379 (29.1)	505 (29.3)	707 (35.5)	753 (35.6)
	Higher	861 (55.0)	1886 (63.5)	265 (20.4)	430 (25.0)	378 (19.0)	564 (26.7)
Occupational status, n (%)	Employed	1062 (67.6)	2291 (76.9)	723 (55.9)	1134 (66.2)	1125 (56.7)	1471 (69.5)
	Not in working	334 (21.3)	470 (15.8)	221 (17.1)	209 (12.2)	261 (13.1)	224 (10.6)
	Unemployed	113 (7.2)	140 (4.7)	224 (17.3)	218 (12.7)	372 (18.7)	274 (12.9)
	Incapacitated	62 (3.9)	78 (2.6)	126 (9.7)	152 (8.9)	227 (11.4)	147 (6.9)
Occupational n (%)	Elementary	34 (2.4)	43 (1.5)	140 (12.3)	149 (9.7)	155 (8.7)	110 (5.6)
	Lower	276 (19.1)	378 (13.3)	448 (39.2)	488 (31.8)	689 (38.6)	635 (32.3)
	Medium	351 (24.3)	653 (23.0)	332 (29.1)	499 (32.5)	625 (35.0)	703 (35.8)
	Higher	784 (54.3)	1768 (62.2)	222 (19.4)	398 (25.9)	318 (17.8)	516 (26.3)
BMI (kg/m^2), mean (SD)		24.9 (4.6)	24.7 (4.0)	26.4 (5.1)	26.2 (4.6)	27.9 (5.9)	27.7 (5.2)
Waist-to-hip ratio, mean (SD)		0.9 (0.1)	0.9 (0.1)	0.9 (0.1)	0.9 (0.1)	0.9 (0.1)	0.9 (0.1)
Waist circumference (cm), mean (SD)		89.2 (14.0)	89.4 (12.3)	92.2 (13.8)	91.5 (12.4)	93.3 (15.0)	92.8 (13.1)
Obesity, n (%)		203 (12.7)	265 (8.9)	465 (35.5)	572 (33.1)	636 (31.6)	595 (27.9)
Central obesity, n (%)		849 (53.1)	1610 (53.8)	902 (68.9)	1237 (71.6)	1289 (64.1)	1415 (66.5)
B		Ghanaian		Turkish		Moroccan	
		Without follow-up	With follow-up	Without follow-up	With follow-up	Without follow-up	With follow-up
		n = 1684	*n = 901*	*n = 2644*	*n = 1099*	*n = 2434*	*n = 1632*

Age (years), median [IQR]		46.0 [36.0, 53.0]	47.0 [40.0, 53.0]	41.0 [29.0, 49.0]	43.0 [33.0, 50.0]	38.0 [27.0, 50.0]	43.0 [34.0, 51.0]
Sex (women), n (%)		1032 (61.3)	544 (60.4)	1454 (55.0)	574 (52.2)	1564 (64.3)	914 (56.0)
Hypertension, n (%)		883 (52.4)	457 (50.9)	683 (25.9)	254 (23.1)	418 (17.2)	329 (20.2)
Current smoker, n (%)		61 (4.1)	42 (5.0)	927 (36.8)	313 (29.4)	363 (15.7)	161 (10.2)
Diabetes mellitus, n (%)		164 (9.8)	65 (7.2)	227 (8.6)	70 (6.4)	251 (10.4)	148 (9.1)
Lipid-lowering medication, n (%)		142 (8.4)	50 (5.5)	266 (10.1)	81 (7.4)	168 (6.9)	107 (6.6)
eGFR (ml/min/1.73 m^2), mean (SD)		105.4 (20.2)	102.1 (18.8)	108.0 (14.9)	106.1 (13.6)	111.1 (15.1)	107.5 (14.3)
History of CVD, n (%)		20 (1.4)	8 (1.0)	37 (1.5)	13 (1.2)	22 (1.0)	12 (0.8)
Educational level, n (%)	Never	421 (28.7)	237 (28.6)	859 (34.2)	274 (25.8)	735 (32.1)	469 (29.8)
	Lower	587 (40.1)	330 (39.9)	638 (25.4)	250 (23.6)	393 (17.2)	301 (19.1)
	Intermediate	374 (25.5)	203 (24.5)	705 (28.1)	314 (29.6)	797 (34.8)	497 (31.6)
	Higher	83 (5.7)	58 (7.0)	310 (12.3)	222 (20.9)	365 (15.9)	308 (19.6)
Occupational status, n (%)	Employed	852 (58.4)	515 (62.5)	1242 (49.9)	628 (59.5)	1033 (45.1)	861 (54.8)
	Not in working	116 (8.0)	40 (4.9)	630 (25.3)	192 (18.2)	693 (30.2)	362 (23.0)
	Unemployed	354 (24.3)	203 (24.6)	380 (15.3)	156 (14.8)	378 (16.5)	226 (14.4)
	Incapacitated	136 (9.3)	66 (8.0)	235 (9.4)	80 (7.6)	188 (8.2)	123 (7.8)
Occupational n (%)	Elementary	805 (63.9)	447 (63.6)	390 (21.1)	147 (17.0)	301 (18.5)	203 (17.1)
	Lower	295 (23.4)	164 (23.3)	793 (42.9)	316 (36.5)	585 (35.9)	393 (33.1)
	Medium	112 (8.9)	62 (8.8)	445 (24.1)	220 (25.4)	463 (28.4)	350 (29.5)
	Higher	47 (3.7)	30 (4.3)	219 (11.9)	183 (21.1)	281 (17.2)	242 (20.4)
BMI (kg/m^2), mean (SD)		28.5 (5.2)	28.3 (4.4)	28.8 (6.0)	28.0 (5.2)	27.5 (5.5)	27.6 (4.8)
Waist-to-hip ratio, mean (SD)		0.9 (0.1)	0.9 (0.1)	0.9 (0.1)	0.9 (0.1)	0.9 (0.1)	0.9 (0.1)
Waist circumference (cm), mean (SD)		93.2 (13.1)	92.8 (11.4)	95.0 (14.5)	93.4 (13.1)	92.7 (14.1)	93.9 (12.6)
Obesity, n (%)		581 (34.5)	291 (32.3)	991 (37.5)	323 (29.4)	735 (30.2)	440 (27.0)
Central obesity, n (%)		1140 (67.8)	625 (69.4)	1860 (70.4)	741 (67.4)	1608 (66.1)	1127 (69.1)

IQR = interquartile range. SD standard deviation. BMI = body mass index. eGFR = estimated glomerular filtration rate. CVD = cardiovascular diseases. Obesity was defined as a BMI of ≥27.5 kg/m2 for the South-Asian Surinamese and ≥30 kg/m2 for the other ethnic groups. Central obesity was defined as a waist circumference of ≥80 cm for women, ≥90 cm for South-Asian Surinamese men and ≥95 cm for non-South-Asian Surinamese men. Hypertension was defined as RR ≥140/90 mmHg or use of antihypertensive medication. Diabetes mellitus was defined as glucose ≥7 mmol/l or use of glucose-lowering medication. History of CVD was defined as self-reported stroke, myocardial infarction, and coronary or peripheral revascularization.

Supplementary Table 2: Detailed linear mixed models of ethnic differences in BMI in younger and older participants. Model 1 was adjusted for age and sex, Model 2 was adjusted for age, sex, educational level, occupational status and occupational level.

		**Younger (< 50 years)**						**Older (≥ 50 years)**					
		Model1			Model 2			Model 1			Model 2		
		β	95% CI		β	95% CI		β	95% CI		β	95% CI	
Intercept		17.86*	17.49	18.23	21.10*	20.47	21.74	21.24*	20.16	22.32	25.60*	24.21	26.99
Age (years)		0.15*	0.15	0.16	0.13*	0.12	0.14	0.06*	0.04	0.08	0.02*	0.00	0.04
Sex (women)		0.67*	0.51	0.83	0.35*	0.18	0.53	2.55*	2.35	2.75	2.01*	1.79	2.24
Educational level	Lower				-0.52*	-0.86	-0.18				-0.67*	-1.02	-0.33
(ref = never)	Intermediate				-0.71*	-1.06	-0.36				-0.93*	-1.33	-0.53
	Higher				-1.40*	-1.82	-0.97				-1.45*	-1.97	-0.94
Occupational status	Not in working				0.02	-0.31	0.36				0.44*	0.08	0.80
(ref = employed)	Unemployed				0.29*	0.02	0.57				0.38*	0.05	0.70
	Incapacitated				0.63*	0.19	1.06				1.18*	0.83	1.53
Occupational level	Lower				-0.79*	-1.11	-0.47				-0.56*	-0.92	-0.21
(ref = elementary)	Medium				-0.68*	-1.03	-0.33				-0.60*	-1.02	-0.19
	Higher				-1.07*	-1.49	-0.65				-1.16*	-1.68	-0.63
Ethnic differences in	Dutch	Ref	Ref	Ref	Ref	Ref	Ref	Ref	Ref	Ref	Ref	Ref	Ref
BMI at baseline	SA Surinamese	1.82*	1.52	2.11	1.31*	1.00	1.62	1.14*	0.80	1.47	0.34	-0.03	0.70
	A Surinamese	2.91*	2.63	3.20	2.47*	2.16	2.77	2.48*	2.19	2.77	1.92*	1.60	2.23
	Ghanaian	3.80*	3.50	4.11	2.59*	2.20	2.97	3.46*	3.09	3.83	1.93*	1.46	2.41
	Turkish	4.02*	3.76	4.28	2.97*	2.67	3.27	5.29*	4.92	5.67	3.16*	2.69	3.63
	Moroccan	3.11*	2.86	3.37	2.21*	1.91	2.50	3.86*	3.51	4.21	1.54*	1.06	2.02
Visit (0 = baseline. 1 = follow-up)		-0.37*	-0.49	-0.24	-0.17*	-0.30	-0.04	-0.23*	-0.38	-0.08	-0.02*	-0.16	0.19
Additional increase	Dutch	Ref	Ref	Ref	Ref	Ref	Ref	Ref	Ref	Ref	Ref	Ref	Ref
in BMI at follow-up	SA Surinamese	0.44*	0.26	0.62	0.45*	0.26	0.63	0.02	-0.14	0.19	0.08	-0.09	0.26
compared to the	A Surinamese	0.54*	0.36	0.73	0.50*	0.31	0.69	0.08	-0.06	0.23	0.08	-0.07	0.23
Dutch reference	Ghanaian	0.81*	0.60	1.03	0.78*	0.55	1.02	0.45*	0.23	0.67	0.45*	0.20	0.69
population	Turkish	0.34*	0.15	0.53	0.37*	0.17	0.58	0.47*	0.23	0.70	0.51*	0.24	0.78
	Moroccan	0.44*	0.27	0.61	0.38*	0.19	0.56	0.03	-0.16	0.22	0.14	-0.09	0.37

* = Statistically significant (P<0.05). *CI = confidence interval. ref = reference population. S.A. = South-Asian. A. = African. BMI = body mass index.*

Supplementary Table 3 (BMI): Showing the percentage attenuation-additional increase in BMI compared to the Dutch reference population explained by SEP.

Ethnic Group	Age Group	Model 1 β (95% CI)	Model 2 β (95% CI)	Percentage Attenuation
**Ghanaians**	< 50 yrs	0.81 (0.60, 1.03)	0.80 (0.58, 1.01)	**1.2%**
	> 50 yrs	0.45 (0.23, 0.67)	0.45 (0.23, 0.68)	**0.0%**
**African Surinamese**	< 50 yrs	0.49 (0.33, 0.65)	0.47 (0.31, 0.63)	**4.1%**
	> 50 yrs	0.04 (-0.14, 0.23)	0.02 (-0.17, 0.21)	**50.0%***
**South-Asian Surinamese**	< 50 yrs	0.38 (0.22, 0.54)	0.35 (0.19, 0.51)	**7.9%**
	> 50 yrs	0.05 (-0.12, 0.22)	0.03 (-0.14, 0.21)	**40.0%***
**Turkish**	< 50 yrs	0.39 (0.20, 0.59)	0.35 (0.15, 0.55)	**10.3%**
	> 50 yrs	0.46 (0.23, 0.69)	0.44 (0.21, 0.67)	**4.3%**
**Moroccan**	< 50 yrs	0.17 (0.01, 0.33)	0.14 (-0.02, 0.30)	**17.6%**
	> 50 yrs	0.08 (-0.10, 0.26)	0.03 (-0.15, 0.21)	**62.5%***

*High attenuation percentages in older groups are often due to very small baseline differences (low β values).

Supplementary Table 4 (WC): Showing the percentage attenuation-additional increase in WC compared to the Dutch reference population explained by SEP

Ethnic Group	Age Group	Model 1 β (95% CI)	Model 2 β (95% CI)	Percentage Attenuation
**Ghanaians**	< 50 yrs	1.47 (0.87, 2.06)	1.40 (0.81, 1.99)	**4.8%**

Ethnic Group	Age Group	Model 1 β (95% CI)	Model 2 β (95% CI)	Percentage Attenuation
	> 50 yrs	0.38 (0.05, 0.70)	0.34 (0.02, 0.67)	**10.5%**
**African Surinamese**	< 50 yrs	1.25 (0.81, 1.68)	1.20 (0.77, 1.63)	**4.0%**
	> 50 yrs	-0.06 (-0.32, 0.20)	-0.10 (-0.37, 0.17)	**N/A****
**South-Asian Surinamese**	< 50 yrs	0.69 (0.25, 1.13)	0.64 (0.20, 1.08)	**7.2%**
	> 50 yrs	0.03 (-0.21, 0.27)	0.01 (-0.24, 0.26)	**66.7%***
**Turkish**	< 50 yrs	0.35 (-0.19, 0.88)	0.28 (-0.25, 0.82)	**20.0%**
	> 50 yrs	0.14 (-0.18, 0.46)	0.11 (-0.21, 0.44)	**21.4%**
**Moroccan**	< 50 yrs	0.18 (-0.26, 0.61)	0.12 (-0.31, 0.56)	**33.3%**
	> 50 yrs	-0.01 (-0.26, 0.25)	-0.08 (-0.35, 0.18)	**N/A****

*High attenuation percentages in older groups are often due to very small baseline differences (low β values). ** For these groups, the ethnic difference was already negative (meaning a smaller increase than the Dutch) and became further negative in Model 2, indicating that adjusting for SEP actually increased the observed gap.

Supplementary Table 5: Ethnic differences in changes in BMI over time compared to the Dutch reference group as derived from linear mixed models, for men (A) and women (B). Model 1 was adjusted for age. Model 2 was adjusted for age, educational level, occupational status and occupational level.

A	Men < 50 years		Men ≥ 50 years	
	Model 1	Model 2	Model 1	Model 2

		β	95% CI		P-value	β	95% CI		P-value	β	95% CI		P-value	β	95% CI		P-value
Difference in BMI between baseline and follow-up in Dutch reference population		0.48	0.47	0.49	<0.001	0.46	0.45	0.47	<0.001	0.05	0.04	0.06	<0.001	0.05	0.04	0.06	<0.001
Additional increase in BMI at follow-up compared to Dutch reference population	Dutch	Ref	Ref	Ref	Ref	Ref	Ref	Ref	Ref	Ref	Ref	Ref	Ref	Ref	Ref	Ref	Ref
	SA Surinamese	0.46	0.23	0.68	<0.001	0.42	0.19	0.64	<0.001	0.12	-0.09	0.34	0.253	0.16	-0.06	0.38	0.156
	A Surinamese	0.41	0.16	0.66	0.001	0.40	0.15	0.65	0.002	0.16	-0.03	0.35	0.096	0.16	-0.03	0.36	0.106
	Ghanaian	0.34	0.03	0.65	0.030	0.31	-0.03	0.64	0.071	0.44	0.18	0.71	0.001	0.49	0.20	0.77	0.001
	Turkish	0.46	0.23	0.70	<0.001	0.51	0.27	0.76	<0.001	0.40	0.12	0.68	0.005	0.41	0.12	0.71	0.006
	Moroccan	0.25	0.03	0.47	0.025	0.26	0.04	0.49	0.021	0.24	0.00	0.47	0.046	0.30	0.05	0.55	0.020

B		Women < 50 years								Women ≥ 50 years							
		Model 1				Model 2				Model 1				Model 2			
		β	95% CI		P-value	β	95% CI		P-value	β	95% CI		P-value	β	95% CI		P-value
Difference in BMI between baseline and follow-up in Dutch reference population		0.71	0.70	0.72	<0.001	0.75	0.74	0.77	<0.001	0.21	0.20	0.22	<0.001	0.21	0.20	0.23	<0.001
Additional increase in BMI at follow-up compared to Dutch reference population	Dutch	0.44	0.17	0.71	0.001	0.48	0.19	0.76	0.001	-0.06	-0.30	0.18	0.642	0.01	-0.24	0.27	0.916
	SA Surinamese	0.58	0.33	0.84	<0.001	0.50	0.23	0.77	<0.001	0.00	-0.21	0.21	0.999	0.00	-0.22	0.22	0.976
	A Surinamese	0.99	0.69	1.29	<0.001	0.96	0.63	1.29	<0.001	0.48	0.14	0.82	0.006	0.41	0.02	0.81	0.041
	Ghanaian	0.24	-0.04	0.53	0.095	0.22	-0.09	0.53	0.165	0.52	0.16	0.88	0.005	0.70	0.24	1.16	0.003
	Turkish	0.57	0.32	0.82	<0.001	0.48	0.20	0.76	0.001	-0.15	-0.44	0.14	0.315	-0.10	-0.53	0.33	0.635
	Moroccan	0.44	0.17	0.71	0.001	0.48	0.19	0.76	0.001	-0.06	-0.30	0.18	0.642	0.01	-0.24	0.27	0.916

CI = confidence interval. ref = reference population. S.A. = South-Asian. A. = African. BMI = body mass index.

Supplementary Table 6: Ethnic differences in changes in waist circumference over time compared to the Dutch reference group as derived from linear mixed models, for men (A) and women (B). Model 1 was adjusted for age. Model 2 was adjusted for age, educational level, occupational status and occupational level.

**A**		**Men < 50 years**								**Men ≥ 50 years**							
		Model 1				Model 2				Model 1				Model 2			
		β	95% CI		P-value	β	95% CI		P-value	β	95% CI		P-value	β	95% CI		P-value
Difference in waist circumference between baseline and follow-up in Dutch reference population		1.07	0.96	1.19	<0.001	0.99	0.88	1.10	<0.001	0.04	-0.05	0.14	0.409	0.12	0.02	0.22	0.019
Additional increase in waist circumference at follow-up compared to Dutch reference population	Dutch	Ref	Ref	Ref	Ref	Ref	Ref	Ref	Ref	Ref	Ref	Ref	Ref	Ref	Ref	Ref	Ref
	SA Surinamese	0.99	0.22	1.75	0.011	0.81	0.04	1.58	0.040	0.01	-0.77	0.79	0.984	0.11	-0.70	0.92	0.786
	A Surinamese	1.35	0.51	2.19	0.002	1.44	0.59	2.28	0.001	0.30	-0.40	0.99	0.406	0.19	-0.52	0.91	0.596
	Ghanaian	0.69	-0.35	1.73	0.192	0.64	-0.48	1.76	0.260	0.61	-0.35	1.57	0.213	0.82	-0.21	1.84	0.119
	Turkish	0.28	-0.51	1.08	0.489	0.51	-0.32	1.33	0.227	-0.29	-1.29	0.72	0.577	-0.33	-1.39	0.74	0.547
	Moroccan	0.34	-0.40	1.08	0.371	0.38	-0.38	1.14	0.323	0.10	-0.76	0.95	0.822	0.19	-0.72	1.09	0.689

B		Women < 50 years								Women ≥ 50 years							
		Model 1				Model 2				Model 1				Model 2			
		β	95% CI		P-value	β	95% CI		P-value	β	95% CI		P-value	β	95% CI		P-value
Difference in waist circumference between baseline and follow-up in Dutch reference population		0.82	0.67	0.98	<0.001	0.93	0.77	1.09	<0.001	-0.04	-0.17	0.08	0.532	-0.02	-0.15	0.11	0.763
Additional increase in waist circumference at follow-up compared to Dutch reference population	Dutch	Ref	Ref	Ref	Ref	Ref	Ref	Ref	Ref	Ref	Ref	Ref	Ref	Ref	Ref	Ref	Ref
	SA Surinamese	0.66	-0.21	1.54	0.137	0.76	-0.17	1.68	0.108	-0.80	-1.60	0.00	0.051	-0.67	-1.52	0.18	0.122
	A Surinamese	1.12	0.29	1.96	0.008	1.07	0.20	1.94	0.015	-0.55	-1.25	0.16	0.128	-0.57	-1.29	0.16	0.124
	Ghanaian	1.91	0.95	2.88	<0.001	1.67	0.59	2.74	0.002	0.41	-0.73	1.54	0.483	0.17	-1.14	1.47	0.803
	Turkish	-1.15	-2.07	-0.23	0.014	-0.91	-1.92	0.11	0.080	-1.21	-2.41	-0.01	0.049	-1.01	-2.53	0.51	0.191
	Moroccan	-0.34	-1.15	0.47	0.406	-0.37	-1.28	0.54	0.430	-1.16	-2.13	-0.19	0.019	-1.12	-2.54	0.29	0.119

CI = confidence interval. ref = reference population. S.A. = South-Asian. A. = African. BMI = body mass index.

Supplementary Table 7: Sensitivity analysis on the effect of imputation on complete cases for BMI.

		Younger (< 50 years)								Older (≥ 50 years)							
		Without imputation				With imputation				Without imputation				With imputation			
		β	95% CI		P-value	β	95% CI		P-value	β	95% CI		P-value	β	95% CI		P-value
Difference in BMI between baseline and follow-up in Dutch reference population		0.61	0.60	0.62	<0.001	0.39	0.37	0.41	<0.001	0.14	0.14	0.15	<0.001	0.06	0.04	0.09	<0.001
Additional increase in BMI at follow-up compared to Dutch reference population	Dutch	Ref	Ref	Ref	Ref	Ref	Ref	Ref	Ref	Ref	Ref	Ref	Ref	Ref	Ref	Ref	Ref
	SA Surinamese	0.44	0.26	0.62	<0.001	0.38	0.01	0.75	0.046	0.02	-0.14	0.19	0.773	0.08	-0.29	0.46	0.665
	A Surinamese	0.54	0.36	0.73	<0.001	0.56	0.20	0.91	0.002	0.08	-0.07	0.22	0.281	-0.05	-0.37	0.28	0.782
	Ghanaian	0.81	0.60	1.03	<0.001	0.52	0.11	0.94	0.013	0.45	0.23	0.67	0.000	0.18	-0.29	0.65	0.449
	Turkish	0.34	0.15	0.53	<0.001	0.29	-0.07	0.65	0.109	0.46	0.23	0.69	0.000	0.15	-0.38	0.68	0.568
	Moroccan	0.44	0.27	0.61	<0.001	0.45	0.11	0.79	0.009	0.03	-0.16	0.22	0.760	-0.04	-0.46	0.39	0.868

Models were adjusted for age and sex. CI = confidence interval. ref = reference population. S.A. = South-Asian. A. = African. WC = waist circumference

Supplementary Table 8: Sensitivity analysis on the effect of imputation on complete cases for waist circumference.

		**Younger (< 50 years)**								**Older (≥ 50 years)**							
		Without imputation				With imputation				Without imputation				With imputation			
		β	95% CI		P-value	β	95% CI		P-value	β	95% CI		P-value	β	95% CI		P-value
Difference in WC between baseline and follow-up in Dutch reference population		0.97	0.90	1.04	<0.001	0.81	0.67	0.94	<0.001	0.01	-0.05	0.07	0.743	-0.60	-0.74	-0.45	<0.001
Additional increase in WC at follow-up compared to Dutch reference population	Dutch	Ref	Ref	Ref	Ref	Ref	Ref	Ref	Ref	Ref	Ref	Ref	Ref	Ref	Ref	Ref	Ref
	SA Surinamese	0.78	0.19	1.37	0.010	0.52	-0.48	1.52	0.308	-0.49	-1.06	0.08	0.089	-0.21	-1.18	0.76	0.670
	A Surinamese	1.17	0.57	1.76	<0.001	1.01	0.04	1.99	0.042	-0.23	-0.72	0.27	0.376	-0.27	-1.14	0.59	0.534
	Ghanaian	1.47	0.76	2.18	<0.001	0.82	-0.20	1.84	0.116	0.49	-0.26	1.25	0.199	0.18	-0.99	1.35	0.765
	Turkish	-0.49	-1.11	0.13	0.123	-0.40	-1.30	0.49	0.374	-0.73	-1.52	0.07	0.072	-0.92	-2.14	0.30	0.137
	Moroccan	-0.09	-0.65	0.47	0.757	0.26	-0.64	1.16	0.566	-0.58	-1.24	0.07	0.080	-0.56	-1.65	0.53	0.313

Models were adjusted for age and sex. CI = confidence interval. ref = reference population. S.A. = South-Asian. A. = African. WC = waist circumference

Supplementary Figure 1: A scatter plot of BMI versus age, showing the non-linear curve inflection point at age 50yrs.

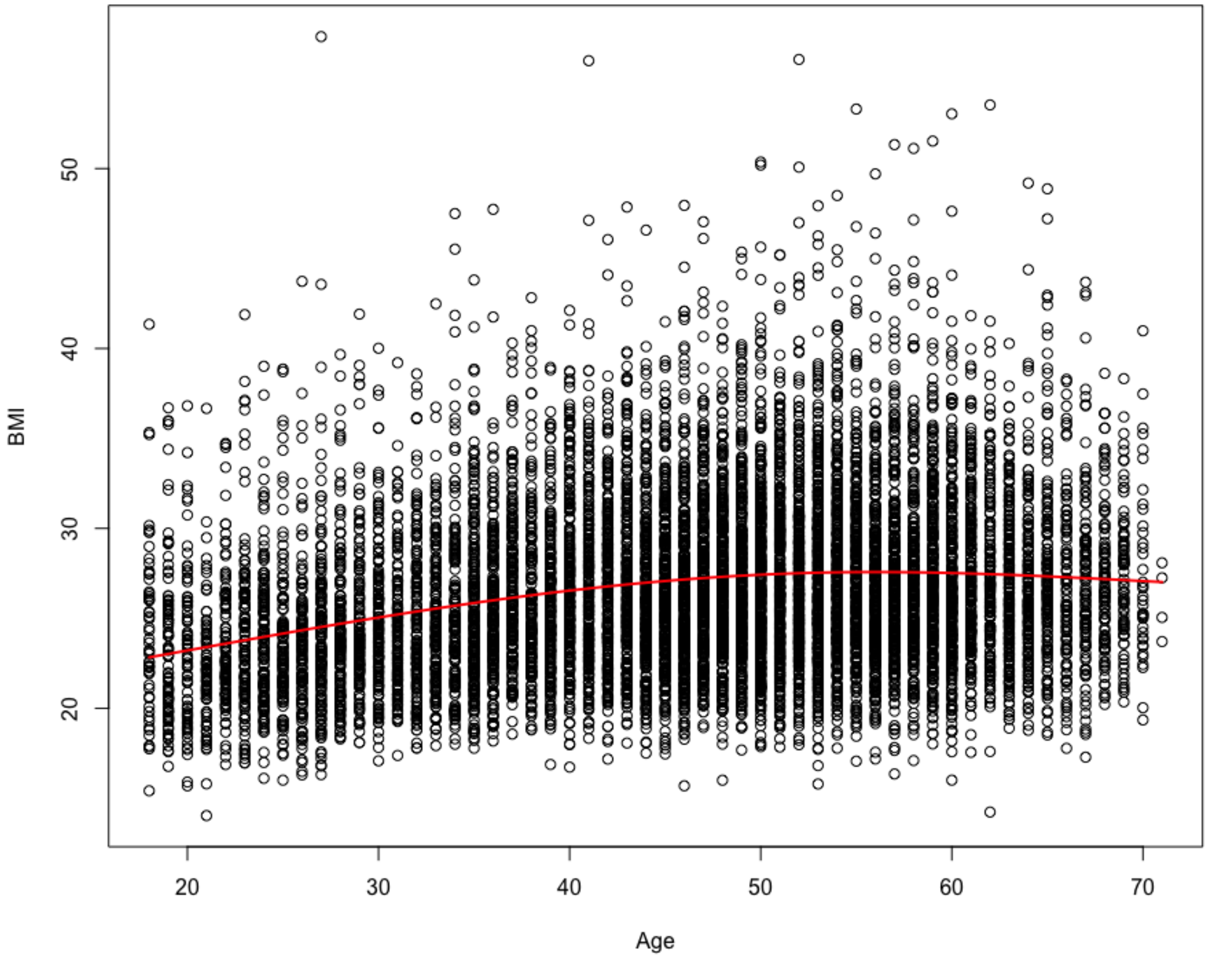

Supplementary Figure 2: Flowchart of the inclusion of participants.

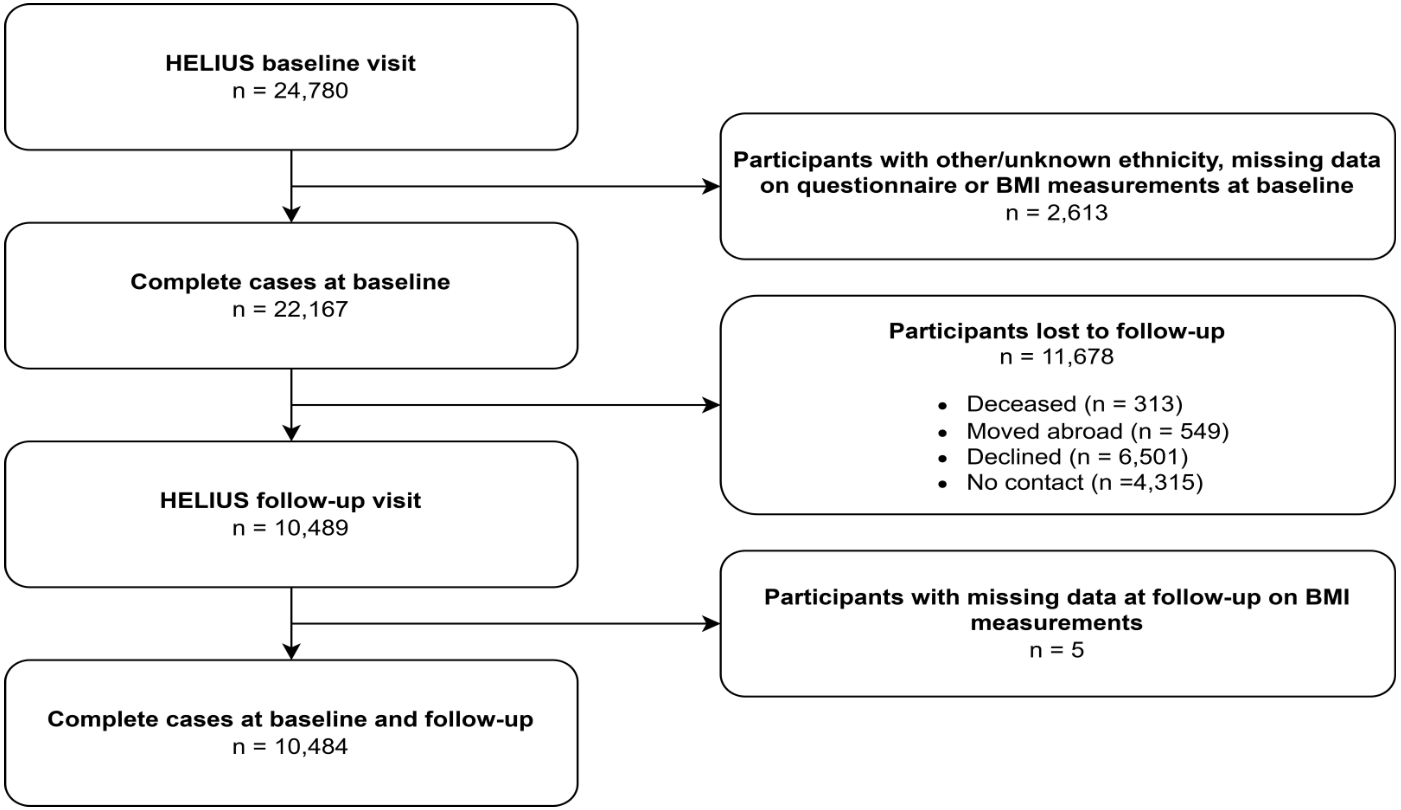

BMI = body mass index. HELIUS = Healthy Life in an Urban setting.

Supplementary Figure 3: Relationship between BMI (kg/m²) and age (years) at baseline and follow-up stratified per sex.

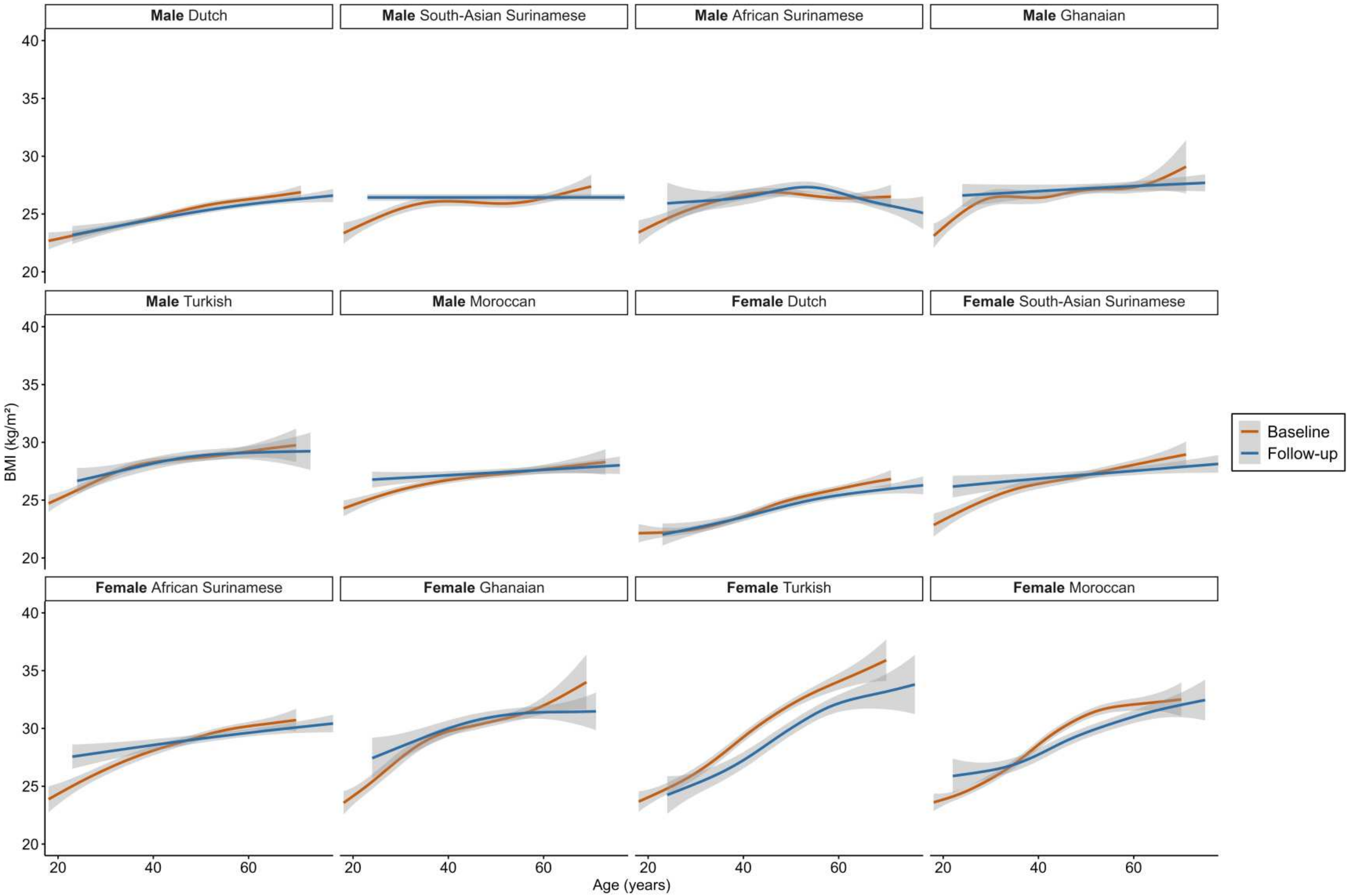

Supplementary Figure 4: Relationship between waist circumference (cm) and age (years) at baseline and follow-up stratified per sex.

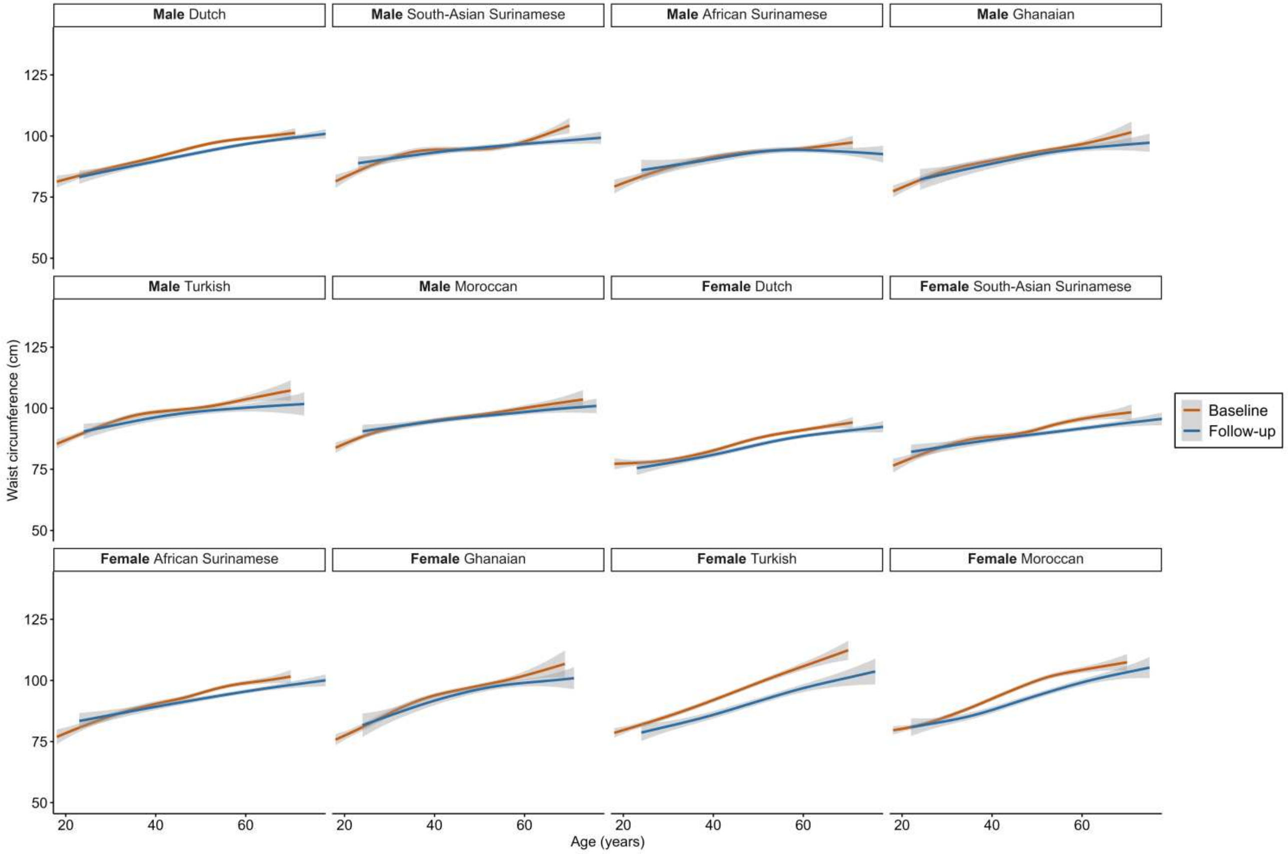

Supplement: Supplementary data [file bmjph-4-2-s001.pdf]
